# Validity and Reliability of the Breast Cancer Screening Beliefs Questionnaire Among Ghanaian Women

**DOI:** 10.1155/ijbc/6682781

**Published:** 2026-05-06

**Authors:** Enoch Teye-Kwadjo

**Affiliations:** ^1^ Department of Psychology, University of Ghana, Legon, Ghana, ug.edu.gh

**Keywords:** attitudes, barriers, breast cancer screening, ESEM, knowledge, mammography, women

## Abstract

**Background:**

Breast cancer screening allows early detection of treatable breast cancer malignancies. However, the use of breast cancer screening among asymptomatic women in Ghana is reported to be generally low. In addition, Ghana does not have a standardized measure to assess and quantify breast cancer knowledge and screening beliefs.

**Methods:**

This study examined the factor structure and internal consistency reliability of the Breast Cancer Screening Beliefs Questionnaire (BCSBQ‐12) in the context of Ghana. A total of 857 women from the Greater Accra Region of Ghana completed the BCSBQ‐12. Exploratory structural equation modelling (ESEM) and traditional confirmatory factor analysis (CFA) were applied to the data via robust maximum likelihood estimation.

**Results:**

The results showed that, compared with the CFA solution, the ESEM solution provided a better fit to the data, with reduced interfactor correlations and adequate internal consistency reliability. The ESEM with target rotation supported the first‐order three‐factor structure proposed by the BCSBQ‐12, indicating the importance of considering breast cancer screening uptake from, at least, three key domains (i.e., *attitudes*, *knowledge,* and *barriers*) in the context of Ghana.

**Conclusions:**

The results provide sound evidence of construct validity and psychometric properties for the use of the BCSBQ‐12 for assessing breast cancer knowledge and screening beliefs among asymptomatic women in Ghana.

## 1. Introduction

Breast cancer deaths can be substantially reduced through screening and early detection [1–3]. Screening allows early detection of breast cancer malignancies or small tumors with fewer nodal metastases that are treatable [4, 5]. Accordingly, breast cancer screening in asymptomatic women through mammography is recommended [6]. Mammographic screening is considered an effective imaging modality for women aged 40–74 years with an average risk for breast cancer [7, 8].

Female breast cancer poses a population health burden in Ghana [9–11]. Although Ghana has a National Strategy for Cancer Control [12, 13], there is no National Breast Cancer Screening Programme in the country [14]. Nonetheless, the month of October each year (i.e., the breast cancer awareness month) is used by healthcare facilities, corporate organizations, higher education institutions, churches, mosques, media houses, nongovernmental organizations, faith‐based organizations, and community‐based organizations in Ghana to undertake massive breast cancer awareness campaigns aimed at encouraging screening and early detection [15–20]. With the aim of providing accurate knowledge about breast cancer through the identification and correction of misconceptions and myths, these campaigns engender favorable attitudes toward breast cancer screening and suggest ways to overcome barriers to breast screening uptake [21, 22]. The campaigns often team up with health facilities to offer free screening opportunities. In addition, the Ghana National Health Insurance Scheme (NHIS) covers the cost of breast cancer screening and treatment for all members of the scheme [23].

However, research in Ghana has revealed that breast cancer knowledge is low among adult women [24, 25] and female students [26], and other research reported that misconceptions emanating from cultural and spiritual beliefs are important barriers to breast cancer care in Ghana [27–29]. Numerous studies have shown that the incidence of breast cancer screening among women in Ghana is generally low [30–33], leading to an increasing incidence of breast cancer among young and premenopausal women in Ghana [34, 35]. However, there is little or no means to assess and quantify breast cancer knowledge and screening beliefs at the community or population level in Ghana because there is no standardized, national measure to carry out such survey research. Standardized measures are needed to undertake population‐based health surveys to identify incidence and prevalence rates and to facilitate comparative analysis.

To address this gap in the breast cancer literature in Ghana, the present study assessed the validity and internal consistency reliability of the Breast Cancer Screening Beliefs Questionnaire (BCSBQ). The BCSBQ is a 13‐item self‐report measure initially developed by Kwok et al. [36] in a Chinese–Australian female population and then validated in African–Australian [37] and Arabic–Australian female populations [38] to assess breast cancer screening uptake in the general population. The BCSBQ‐13 is composed of three dimensions: (a) *attitudes toward health check-ups* (four items), (b) *knowledge and perceptions about breast cancer* (four items), and (c) *barriers to mammography screening practices* (five items). Hereinafter, we refer to the three factors as *attitudes*, *knowledge*, and *barriers*. The *attitudes* dimension assesses a woman′s attitudes toward general health check‐ups in the absence of signs and symptoms of a disease such as breast cancer. The *knowledge* dimension assesses sociocultural beliefs about breast cancer, and the *barriers* dimension explores practical issues perceived by women to hinder the uptake of mammography screening.

The BCSBQ‐13 is therefore a promising measure for assessing breast cancer knowledge and screening beliefs in Ghana. Previous studies elsewhere have reported that the BCSBQ‐13 has good construct validity and reliability [37, 38]. The BCSBQ‐13 was developed via exploratory factor analysis (EFA) ([36]) and validated via confirmatory factor analysis (CFA) ([37, 38]). Although EFA and CFA are important tests, EFA cannot test a hypothesis related to the factor structure of a measure, and CFA has some limitations that make it unsuitable for examining the factor structure of multidimensional measures such as the BCSBQ‐13, whose proposed theoretical structure is a first‐order three‐factor oblique model. For example, the independent cluster model CFA allows indicators to load only on their respective factor, with nontarget loadings set to zero [39–42]. To overcome the limitations associated with the EFA and CFA approaches, the exploratory structural equation modelling (ESEM) approach was used in the present study. ESEM with target rotation integrates the best aspects of EFA and CFA by allowing cross‐loadings on nontarget factors to simultaneously test an a priori model‐based factor structure or hypothesis [41, 43]. In addition, ESEM offers greater flexibility in the specification and estimation of multidimensional factorial structures [40, 41, 44, 45], such as that of the BCSBQ.

In this study, one item in the *barriers* dimension, “I do not want to have a mammogram because I cannot speak English,” was considered not to be contextually relevant in the present study and was deleted from the questionnaire before administration. This is because, unlike in Australia where English is the official language and the native language of most Australians, which can pose a barrier to healthcare access for nonnative users such as African‐, Arabic‐, and Chinese–Australians (i.e., the initial sample for development and validation of the BCSBQ), in Ghana, English serves as the lingua franca. English is the medium of instruction from primary school through university and is widely used in government, commerce, and the legal system. As a result, both healthcare practitioners and patients in Ghana generally communicate in English. In addition, most healthcare practitioners and patients often share the same local languages. Therefore, literacy in English language is neither a requirement nor a barrier to accessing healthcare in Ghana. Viewed from this psychometric perspective, removing the English language item was considered to not compromise the validity of the remaining items. Thus, only 12 items on the BCSBQ scale (hereinafter referred to as the BCSBQ‐12) were used in this study.

**Figure 1:**
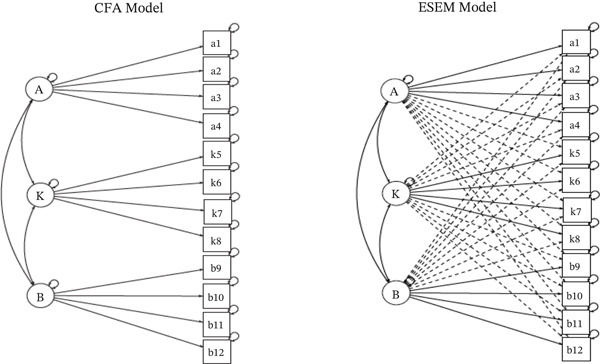
Confirmatory factor analysis (CFA) and exploratory structural equation modelling (ESEM). *Note:* A, K, and B are specific factors. A = attitudes toward health check − ups, K = knowledge about breast cancer, and B = barriers to mammographic screening practices. a1–a4 are indicators of A, k5–k8 are indicators of K, and b9–b12 are indicators of B. Ovals represent latent factors, whereas rectangles represent observed variables. Dotted unidirectional arrows between ovals and rectangles represent cross‐loading. Bidirectional arrows between ovals represent factor correlations/covariances. Curved bidirectional arrows on top of rectangles and ovals represent indicator error and disturbance terms, respectively.

Figure 1 shows the ESEM with target rotation for the BCSBQ‐12. Overall, the objective of the present study was to determine the utility of the BCSBQ‐12 for assessing breast cancer knowledge and screening beliefs in the context of Ghana. The following research questions guided the present study. (a) What is the factor structure of the BCSBQ‐12 in the context of Ghana? (b) What is the internal consistency reliability of the BSCBQ‐12 in the context of Ghana?

## 2. Methods

### 2.1. Participants and Procedure

The data used in the current manuscript were drawn from a substudy (i.e., Screen4BCstudy) of a larger research project, which examined the psychosocial aspects of the breast cancer experience and treatment among Ghanaian women. The substudy is aimed at providing validity and reliability evidence for two behavioral measures—the Champion Breast Cancer Fear Scale (CBCFS) and the BCSBQ. Each measure addressed a distinct research question. For example, the CBCFS data are aimed at assessing how fear of breast cancer influences screening participation by providing evidence of the factorial validity of the CBCFS within the Ghanaian context [46]. Fear is an emotion described as a response to perceived threat or danger. In contrast, the BCSBQ data presented in this paper focuses on cognitive evaluations and perceptions (i.e., attitudes and beliefs) Ghanaian women hold about breast cancer, by examining the factor structure of the BCSBQ within the Ghanaian context.

The participants in the Screen4BCstudy consisted of 857 women recruited from the general population in the Greater Accra Region of Ghana via convenience sampling. Potential participants were approached in communities, churches, universities, corporate institutions, and workplaces and were invited to participate in the study after a brief information session regarding the study′s objectives, anonymity, confidentiality, informed consent, and data collection procedures. Those who expressed interest in the study were recruited. The inclusion criteria were (a) being a woman, (b) being 18 years of age or older, and (c) being able to read and write in English. The exclusion criteria were (a) being a woman diagnosed with breast cancer, (b) being a woman but being younger than 18 years old, and (c) being unable to read and write in English (i.e., the language of the survey questionnaire). Participants provided informed consent and completed a paper‐and‐pencil self‐report questionnaire assessing fear of breast cancer and beliefs about breast cancer screening. The present study was conducted in accordance with the principles of the 1964 Declaration of Helsinki and its later amendments. Ethics approval was granted by the Ethics Committee for the Humanities (Protocol #: ECH104/16‐17), University of Ghana. This study was reported in accordance with the Strengthening the Reporting of Observational Studies in Epidemiology (STROBE) guidelines for observational and cross‐sectional studies. The corresponding checklist was downloaded and completed (refer to the Supporting Information).

### 2.2. Measure

Breast cancer knowledge and screening beliefs were assessed with the BCSBQ‐12 (see Table 1; [36–38]). The 12 items of the BCSBQ (see Table S1 online) address three dimensions (i.e., *attitudes, knowledge,* and *barriers*). All of the items are rated on a 5‐point Likert scale from 1 (*strongly disagree*) to 5 (*strongly agree*), with higher scores indicating more unfavorable attitudes toward general health check‐ups, poorer knowledge about breast cancer, and greater perceived practical barriers to breast cancer screening. Previous psychometric research reported good internal consistency reliabilities for the three dimensions of the BSCBQ in African–Australian women (*α* = 0.84 and *α* = 0.92; [37]), Arabic–Australian women (*α* = 0.81 and *α* = 0.93; [38]), and Chinese–Australian women (*α* = 0.70 and *α* = 0.79; [36]). In this study, participants also provided biographical information on age (in years), educational level, marital status, religious affiliation, employment, and average monthly household income.

**Table 1:** Descriptive statistics of the Breast Cancer Screening Beliefs Questionnaire (BCSBQ^†^).

Item description	*M*	*SD*	Skew	Kurtosis
Attitudes toward health check‐ups (**A**)				
1. If I feel well, it is not necessary to have a health check‐up.	2.29	1.33	0.86	−0.51
2. If I follow a healthy lifestyle such as a balanced diet and regular exercise, I do not feel it is necessary to have a regular health check‐up.	2.29	1.25	0.86	−0.32
3. I see a doctor or have my health check‐up only when I have a health problem.	2.95	1.42	0.03	−1.41
4. If I feel healthy, I do not need to see the doctor.	2.66	1.36	0.39	−1.17
Knowledge and perceptions about breast cancer (**K**)				
5. Breast cancer is like a death sentence; if you get it, you will surely die from it.	2.07	1.14	1.17	0.75
6. Breast cancer cannot be cured; you can only prolong the suffering.	2.16	1.10	0.92	0.31
7. Even if breast cancer is detected early, there is very little a woman can do to reduce the chances of dying from it.	2.18	1.21	0.95	0.01
8. If a woman is fated to get breast cancer, she will get breast cancer; there is nothing she can do to change fate.	1.95	1.07	1.16	0.83
Barriers to mammographic screening practices (**B**)				
9. I am worried that having a mammogram will hurt my breasts.	2.34	1.14	0.65	−0.19
10. It would be difficult to arrange transportation for getting a mammogram.	2.23	1.06	0.72	0.07
11. I do not want to go for a mammogram because I would need to take off my clothes and expose my breasts.	2.27	1.19	0.82	−0.16
12. Having a mammogram is embarrassing.	2.07	1.12	1.09	0.55

*Note*: Rating scale: 1 (*strongly disagree*), 2 (*disagree*), 3 (*neither disagree nor agree*), 4 (*agree*), 5 (*strongly agree*). The bolded letters A, K, and B are the letters used to represent the specific factors in Figure 1.

Abbreviations: *M*, mean; SD, standard deviation.

^†^All of the items on the BCSBQ are available in Kwok et al. [37, 38].

### 2.3. Statistical Analysis

#### 2.3.1. Preliminary Analysis

Item analysis was performed on the data in SPSS (v25). The data (see File S2 online) were first checked for correspondence with parametric assumptions (see Table 1). The normality assumption of the BCSBQ‐12 data was checked via West et al.′s [47] cut‐off criteria of skewness (± 2.00) and kurtosis (± 7.00). All 12 items of the BCSBQ are normally distributed (see Table 2) because the skewness and kurtosis values are within acceptable limits [48–50]. Floor and ceiling effects were also examined. Following previous research [51–53], we considered floor and ceiling effects to be present in the data if > 15% of the participants obtained the highest or lowest possible score on the BCSBQ‐12. There was no significant floor/ceiling effect because only 1.8% (15) of the participants achieved the lowest possible score, whereas 0.4% (3) of the participants obtained the highest possible score. Following this, missing data patterns were evaluated. A few items were missing some data points ranging from 0.1% to 0.2%. Missing value analysis via Little′s MCAR test [54, 55] revealed that the items were missing completely at random, *χ* ^2^ (77) = 39.93, *p* = 0.092. Descriptive statistics were calculated for the demographic variables (see Table 2).

**Table 2:** Demographic characteristics of study participants (*N* = 857).

Variable	Frequency	Percentage	Mean	SD
Age (18–78 years)			26.29	7.15
Gender				
Female	857	100.0		
Marital status				
Never married	660	77.0		
Married	181	21.1		
Separated	16	1.9		
Educational level				
Tertiary	762	88.9		
Secondary	81	9.5		
Basic	14	1.6		
Religious affiliation				
Christianity	792	92.4		
Muslim	60	7.0		
Other	5	0.6		
Employment status				
Working	478	55.8		
Not working	379	44.2		
Average household income (monthly) ^∗^				
< Gh¢1999	398	46.4		
Gh¢2999–Gh¢29994999	182	21.2		
Gh¢5999–Gh¢29997999	120	14.0		
Gh¢8999–Gh¢299910999	67	7.8		
> Gh¢10999	78	9.1		

*Note*:  ^∗^ = 12 responses missing on variable.

#### 2.3.2. Main Analysis

The factor structure of the BCSBQ‐12 was assessed via both CFA and ESEM in Mplus (v7), following the guidelines proposed by methodologists [56–59] (see Table S3 online). In the first phase of the main analysis, an independent cluster model CFA, without cross‐loadings, was fitted to the BCSBQ‐12 data to examine its postulated three‐factor structure via the MLR estimator (see Figure 1). Missing data were handled with the FIML approach. In the second phase of the main analysis, an ESEM was fitted to the BCSBQ‐12 data. In the ESEM, all cross‐loadings were estimated via MLR estimation with a target oblique rotation (see Figure 1). In other words, the ESEM was estimated with all of the cross‐loadings targeted to be as close to zero as possible, as recommended by structural equation modelling methodologists [41, 43, 44]. The indices used to evaluate model fit were the *χ* ^2^ robust test statistic, the comparative fit index (CFI ≥ 0.90 is acceptable fit; ≥ 0.95 is good fit), the root mean square error of approximation (RMSEA ≤ 0.08 is acceptable fit; ≤ 0.06 is good fit) with a 90% confidence interval, and the standardized root mean square residual (SRMR ≤ 0.08 is good fit; [60, 61]). Following previous research [56, 57, 59, 62], the CFA and ESEM solutions were compared via the Satorra–Bentler scaled chi‐square test statistic (*S* − *B* *Δ* *χ* ^2^), change in CFI (*Δ*CFI ≤ 0.010) and changes in RMSEA (*Δ*RMSEA ≤ 0.015) and SRMR (*Δ*SRMR ≤ 0.025). Changes in CFI, RMSEA, and SRMR that are consistent with their associated cut‐off values require that the more parsimonious model be retained [63, 64]. In addition, each item′s measurement quality (parameter estimates) was examined through inspection of standardized factor loadings (*λ* > 0.32; [65, 66]), standardized item uniqueness (> 0.10 but < 0.90), factor correlations, and tolerance levels of cross‐loadings (*λ* > 0.32). Model‐based composite reliabilities of the three dimensions (i.e., *attitudes*, *knowledge*, and *barriers*) and the global factor (i.e., BCSBQ‐12) for both the CFA and ESEM solutions were calculated via McDonald′s omega (*ω*) coefficient [67, 68].

## 3. Results

### 3.1. Sample Characteristics

Table 2 presents the descriptive characteristics of the sample. The sample ranged in age from 18–78 years (*M* = 26.29, SD = 7.15). In terms of educational level, the majority (88.9%) reported having tertiary degrees. As shown in Table 2, the majority of the participants reported having tertiary education (88.9%), being never married (77.0), and working full time (55.8%).

### 3.2. Percentage of Participants Agreeing With BCSBQ Items

Table 3 summarizes the percentage of participants′ responses about breast cancer screening beliefs. Some of the questionnaire responses were combined for ease of reading and understanding. As can be seen in Table 3, most of the respondents (70.5%) strongly disagreed or disagreed with the statement that if a person feels well, they should not submit themselves to a health check‐up. In addition, a majority of the respondents (74.9%) strongly disagreed or disagreed with the statement that breast cancer is like a death sentence, with no opportunity of surviving it. Similarly, about 73.9% of the respondents strongly disagreed or disagreed with the statement that having a mammogram is embarrassing, with nearly 60% (59.9) of them strongly disagreeing or disagreeing with the statement that having a mammogram will hurt their breasts.

**Table 3:** Percentage of participants agreeing with Breast Cancer Screening Beliefs Questionnaire (BCSBQ), items (*N* = 857).

Item description	*N*	Strongly disagree or disagree, *n* (%)	Neither agree nor disagree, *n* (%)	Strongly agree or agree, *n* (%)
Attitudes toward health check‐ups				
1. If I feel well, it is not necessary to have a health check‐up.	857	604 (70.5)	59 (6.9)	194 (22.6)
2. If I follow a healthy lifestyle such as a balanced diet and regular exercise, I do not feel it is necessary to have a regular health check‐up.	855^†^	600 (70.2)	79 (9.2)	176 (20.5)
3. I see a doctor or have my health check‐up only when I have a health problem.	856^†^	395 (46.1)	83 (9.7)	378 (44.1)
4. If I feel healthy, I do not need to see the doctor.	856^†^	485 (56.6)	88 (10.3)	283 (33.0)
Knowledge and perceptions about breast cancer				
13. Breast cancer is like a death sentence; if you get it, you will surely die from it.	856^†^	642 (74.9)	116 (13.5)	98 (11.4)
14. Breast cancer cannot be cured; you can only prolong the suffering.	857	591 (69.0)	169 (19.7)	97 (11.3)
15. Even if breast cancer is detected early, there is very little a woman can do to reduce the chances of dying from it.	857	604 (70.5)	118 (13.8)	135 (15.8)
16. If a woman is fated to get breast cancer, she will get breast cancer; there is nothing she can do to change fate.	857	654 (76.3)	123 (14.4)	80 (9.3)
Barriers to mammographic screening practices				
17. I am worried that having a mammogram will hurt my breasts.	855^†^	513 (59.9)	221 (25.8)	121 (14.1)
18. It would be difficult to arrange transportation for getting a mammogram.	855^†^	552 (64.4)	207 (24.2)	96 (11.2)
19. I do not want to go for a mammogram because I would need to take off my clothes and expose my breasts.	857	566 (66.0)	154 (18.0)	137 (16.0)
20. Having a mammogram is embarrassing.	856^†^	633 (73.9)	122 (14.2)	101 (11.8)

*Note*: BCSBQ items are rated from 1 (*strongly disagree*), 2 (*disagree*), 3 (*neither disagree nor agree*), 4 (*agree*), to 5 (*strongly agree*) with total scores between 12 and 60. Higher scores indicate more unfavorable attitudes toward general health check‐ups, poorer knowledge about breast cancer, and greater perceived practical barriers to breast cancer screening. For ease of reading “strongly disagree” and “disagree” response categories were combined, whereas “agree” and “strongly agree” categories were combined.

^†^Items with missing data points.

### 3.3. Factor Structure of BSCBQ‐12

Table 4 shows the model fit statistics for the CFA and ESEM of the BCSBQ‐12. Table 4 shows that both the CFA model, *χ* ^2^ (51) = 240.02, *p* < 0.001, CFI = 0.911, SRMR = 0.046, RMSEA = 0.066, 90% CI (0.058, 0.074) and the ESEM, *χ* ^2^(33) = 163.08, *p* < 0.001, CFI = 0.939, SRMR = 0.031, RMSEA = 0.068, 90% CI (0.058, 0.078), demonstrated adequate fit to the data. However, an inspection of the model fit statistics shows that the ESEM solution provided a significantly better fit to the data than did the CFA solution. That is, the ESEM solution resulted in a substantial improvement in model fit, *Δ* *χ* ^2^ = 77.33 (*p* < 0.01), *Δ*df = 18, *Δ*CFI = +0.028, *Δ*SRMR = −0.015, and *Δ*RMSEA = 0.002, relative to the CFA solution.

**Table 4:** Model fit statistics for the Breast Cancer Screening Beliefs Questionnaire (BCSBQ‐12).

Model	*χ* ^2^	df	CFI	SRMR	RMSEA (CI_90%_)	*Δχ* ^2^	*Δdf*	*Δ*CFI	*Δ*SRMR	*Δ*RMSEA
CFA	240.02 ^∗∗∗^	51	0.911	0.046	0.066 (0.058, 0.074)					
ESEM	163.08 ^∗∗∗^	33	0.939	0.031	0.068 (0.058, 0.078)	77.33 ^∗∗^	18	+0.028	−0.015	+0.002

*Note*: *Δ* = change in the statistic.

Abbreviations: CI_90%_, 90% confidence interval of the RMSEA; CFA, confirmatory factor analysis; CFI, comparative fit index; df, degrees of freedom; ESEM, exploratory structural equation modelling; RMSEA, root mean square error of approximation; SRMR, standardized root mean square residual;  *X* ^2^, chi‐square.

^∗∗∗^ *p* < 0.01.

### 3.4. Factor Loading

Table 5 presents the factor loadings of the CFA and ESEM solutions of the BCSBQ‐12. Table 5 shows that all of the items loaded significantly on their respective target factor (i.e., their a priori theoretical latent factor) in both solutions. As shown in Table 5, standardized factor loadings were slightly high in both the CFA (|*λ*| = 0.534–0.806, Med_*λ*_ = 0.663) and ESEM (|*λ*| = 0.283–0.829, Med_*λ*_ = 0.662) solutions. The pattern of loading between the two solutions was similar because the differences in loading were small. Specifically, factor loadings for the *attitudes* dimension in the CFA solution (|*λ*| = 0.534–0.806, Med_*λ*_ = 0.693) were similar to those for the *attitudes* dimension in the ESEM solution (|*λ*| = 0.552–0.800, Med_*λ*_ = 0.690). The factor loadings for the *knowledge* dimension in the CFA solution (|*λ*| = 0.613–0.741, Med_*λ*_ = 0.676) were similar to those of the ESEM solution (|*λ*| = 0.586–0.681, Med_*λ*_ = 0.642). Furthermore, the factor loadings on the *barriers* dimension were slightly higher for the CFA solution (|*λ*| = 0.583–0.692, Med_*λ*_ = 0.636) than for the ESEM solution (|*λ*| = 0.283–0.829, Med_*λ*_ = 0.598). As expected in the ESEM solution, there were nontarget cross‐loadings between some items that were conceptually related. Two items on the *attitudes* dimension had significant but small cross‐loadings on the *knowledge* dimension. Furthermore, three items on the *knowledge* dimension were also cross‐loaded on the *barriers* dimension (see Table 5). In general, the cross‐loadings were small and within tolerance levels [65, 66], providing support for the ESEM.

**Table 5:** Standardized factor loadings and uniqueness for the three‐factor CFA and ESEM models of the BCSBQ‐12.

Item description attitudes toward health check‐ups (*A*)	CFA model			ESEM model						
	*λ*	SE	*δ*	A (*λ*)	SE	K (*λ*)	SE	B (*λ*)	SE	*δ*
				
1. If I feel well, it is not necessary to have a health check‐up.	**0.746**	0.029	0.443	**0.723**	0.035	0.042	0.041	0.005	0.039	0.455
2. If I follow a healthy lifestyle such as a balanced diet and regular exercise, I do not feel it is necessary to have a regular health check‐up.	**0.806**	0.027	0.350	**0.800**	0.037	−0.005	0.040	0.020	0.035	0.354
3. I see a doctor or have my health check‐up only when I have a health problem.	**0.534**	0.038	0.715	**0.552**	0.046	−0.044	0.051	0.019	0.046	0.704
4. If I feel healthy, I do not need to see the doctor.	**0.640**	0.036	0.590	**0.656**	0.043	0.011	0.044	−0.039	0.040	0.578
*ω*	**0.** *780*			**0.** *780*						
Knowledge and perceptions about breast cancer (**K**)										
5. Breast cancer is like a death sentence; if you get it, you will surely die from it.	**0.690**	0.029	0.524	0.041	0.030	**0.616**	0.054	0.080	0.048	0.540
6. Breast cancer cannot be cured; you can only prolong the suffering.	**0.741**	0.031	0.451	0.034	0.032	**0.681**	0.061	0.045	0.049	0.484
7. Even if breast cancer is detected early, there is very little a woman can do to reduce the chances of dying from it.	**0.661**	0.031	0.564	−0.075	0.028	**0.667**	0.050	0.058	0.044	0.535
8. If a woman is fated to get breast cancer, she will get breast cancer; there is nothing she can do to change fate.	**0.613**	0.036	0.624	0.115	0.035	**0.586**	0.052	0.004	0.045	0.599
*ω*	*0.772*					*0.733*				
Barriers to mammography screening practices (**B**)										
9. I am worried that having a mammogram will hurt my breasts.	**0.608**	0.043	0.630	−0.021	0.031	0.247	0.068	**0.388**	0.065	0.687
10. It would be difficult to arrange transportation for getting a mammogram.	**0.583**	0.050	0.660	0.011	0.034	0.346	0.069	**0.283**	0.067	0.685
11. I do not want to go for a mammogram because I would need to take off my clothes and expose my breasts.	**0.664**	0.048	0.559	−0.010	0.022	−0.132	0.053	**0.829**	0.075	0.423
12. Having a mammogram is embarrassing.	**0.692**	0.043	0.520	0.020	0.022	−0.093	0.056	**0.808**	0.070	0.417
*ω*	**0.** *732*							**0.** *687*		

*Note*: Target factor loadings are in bold, *λ* = standardized factor loadings, *δ* = standardized item uniqueness, *ω* = omega model − based composite reliability are italicized, nonsignificant cross‐loadings (*p* > 0.05) are underlined, *A* = attitudes toward health check − ups, *K* = knowledge and perceptions about breast cancer, *B* = barriers to mammography screening practices.

Abbreviations: CFA, confirmatory factor analysis; ESEM, exploratory structural equation modelling; SE, standard error.

### 3.5. Factor Correlations

Table 6 presents the latent factor correlations for the BCSBQ‐12. Table 6 shows that the three factors were positively and moderately correlated in both the CFA and ESEM solutions. Inspection of Table 6 indicates that the CFA factor correlations were much greater (|*r*| = 0.281–0.668, Med_*r*_ = 0.353) than those of the ESEM solution with target rotation (|*r*| = 0.253–0.568, Med_*r*_ = 0.304). For example, the positive correlation between the *attitudes* and *knowledge* dimensions was (*r* = 0.353) in the CFA solution. However, the correlation between those two dimensions was reduced to (*r* = 0.304) in the ESEM solution. In addition, the positive correlation between the *knowledge* and *barriers* dimensions was (*r* = 0.668) in the CFA solution but was much lower (*r* = 0.568) in the ESEM solution (see Table 6). An ESEM solution is considered significantly better than a CFA solution if factor correlations are much lower in the ESEM than they are in the CFA model [44, 58, 62, 69].

**Table 6:** Standardized factor correlations for the CFA (below the diagonal) and ESEM (above the diagonal).

Variable	1	2	3
1. Attitudes toward health check‐ups		0.304 ^∗∗∗^	0.253 ^∗∗∗^
2. Knowledge and perceptions about breast cancer	0.353 ^∗∗∗^		0.568 ^∗∗∗^
3. Barriers to mammography screening practices	0.281 ^∗∗∗^	0.668 ^∗∗∗^	

Abbreviations: CFA, confirmatory factor analysis; ESEM, exploratory structural equation modelling.

^∗∗∗^ *p* < 0.001, two‐tail.

### 3.6. Internal Consistency Reliability

The estimates of composite reliability, computed from the standardized factor loadings, for the three well‐defined factors of the BCSBQ‐12 are displayed in Table 5. As shown in Table 5, the score reliability estimates for each dimension of the three‐factor CFA model (*ω* = 0.732–0.780, Med_*ω*_ = 0.772) and the three‐factor ESEM (*ω* = 0.687–0.780, Med_*ω*_ = 0.733) were adequate. The global BCSBQ‐12 scale demonstrated high internal consistency reliability in both the CFA (*ω* = 0.906) and ESEM (*ω* = 0.893) models. Overall, the results support the superiority of the three‐factor ESEM solution, as it offers a much better representation of the BCSBQ‐12 than does the CFA model, which is based on the ESEM′s better fit indices, reduced/lower factor correlations that are unbiased, and its well‐defined factors with adequate internal consistency reliability.

## 4. Discussion

This study is aimed at providing sound validity and reliability evidence for the use of the BCSBQ (BCSBQ‐12) among women in Ghana. Overall, the results from both the CFA and ESEM provided support for the proposed tripartite structure (three‐factor structure) of the BCSBQ‐12 [37, 38], with the ESEM demonstrating a better fit to the data than did the CFA model. These results suggest that, compared with CFA, ESEM seems to be a more appropriate and robust statistical technique for examining the proposed three‐factor structure of the BCSBQ‐12. In terms of reliability using McDonald′s omega coefficient (*ω*), each of the three well‐defined factors of the BCSBQ‐12 (i.e., *attitudes toward health check-ups, knowledge and perceptions about the breast,* and *barriers to mammography screening practices*) demonstrated adequate internal consistency reliability. The global BCSBQ‐12 factor also showed high reliability.

In other words, attitudes toward health check‐ups, knowledge and perceptions about breast, and barriers to mammography screening practices were found to be salient for breast cancer screening participation in the Ghanaian context, which is consistent with the results of previous research in African–Australian women [37], Arabic–Australian women [38], and Chinese–Australian women [36]. Other studies have shown that knowledge about breast cancer, breast cancer‐related attitudes and beliefs, and barriers are important determinants of mammography screening participation in Chinese–Australian women [70], accentuating the tripartite structure of the BCSBQ‐12. Perceived barriers to mammography screening and attitudes toward health check‐ups were also reported to be critical for breast cancer screening uptake [71]. Recent research in Ghana also revealed evidence that having good knowledge of breast cancer was positively associated with mammography screening uptake [72]. Correspondingly, the present results indicate that the BCSBQ‐12 is a promising measure for assessing breast cancer screening knowledge and beliefs among women in Ghana.

Importantly, the BCSBQ‐12 is anchored in knowledge, attitudes, and practice (KAP) theory [73, 74], which is widely used to understand public health problems such as breast cancer [75–77]. Consistent with the KAP framework, the tripartite structure of the BCSBQ‐12 (i.e., *attitudes*, *knowledge*, and *barriers*) is a crucial determinant of health behavior change, such that having accurate knowledge about breast cancer could enhance favorable attitudes toward breast cancer screening, which, in turn, may reduce perceived barriers to breast cancer screening practices. Thus, the use of the BCSBQ‐12 in Ghana may help identify inaccurate knowledge about breast cancer, unfavorable attitudes toward breast cancer screening participation, and perceived barriers to mammography screening practices among women in Ghana.

To our knowledge, this study is the first to use the ESEM framework to examine the postulated factor structure of the BCSBQ‐12. The results, thus, provide important insights into the functioning of the 12 items of the BCSBQ. For example, the ESEM results revealed that all of the items loaded strongly and significantly on their respective factor, with very few items cross‐loading significantly but small on nontarget factors. In other words, the nontarget loadings are relatively smaller than the target loadings, with most of them being nonsignificant. The cross‐loadings in the ESEM solution show that the BCSBQ‐12 items demonstrated potential theoretical overlaps with nontarget factors. This is an important finding, given that this information has not been previously explored relative to the BCSBQ. Consequently, this study makes an important contribution to the breast cancer literature.

Taken together, the ESEM with target rotation provided a better representation of the BCSBQ‐12 data than did the CFA solution, underscoring the importance of comparing CFA and ESEM solutions relative to model fit indices and parameter estimates, which is consistent with the procedures outlined by various structural equation modelling methodologists [56–59]. In this study, the ESEM had improved model fit indices, reduced interfactor correlations, and adequate construct reliability. The reduced interfactor correlations in the ESEM solution indicate that the three factors (i.e., *attitudes*, *knowledge,* and *barriers*) are more distinct (i.e., have enough nonshared variance), despite having enough shared variance (i.e., first‐order three‐factor oblique model). Overall, the present results provide support for the three‐factor structure and psychometric properties of the BCSBQ‐12 in the context of Ghana.

### 4.1. Limitations and Strengths

As with all research, the present study is not without limitations. First, the BCSBQ‐12 is a self‐report measure; therefore, the results of this study are based on self‐reported data. Although very popular in social science research, self‐report measures are known to suffer from response biases such as socially desirable responses. Second, the sample was recruited from the Greater Accra Region of Ghana, the political and economic capital of Ghana and only one of the 16 administrative regions in Ghana. Moreover, our focus on restricting participation in this study to Ghanaian women who could read and write in English (as contained in our inclusion criteria), which was considered necessary to avoid translating the BCSBQ‐12 into a local language, may have introduced some degree of selection bias (i.e., being biased toward women with literacy in English). We recognize that not all Ghanaian woman in the Greater Accra Region who wish to participate in breast cancer screening are literate in English. Apart from English literacy, the characteristics of the present sample may not differ significantly from those of women in other administrative regions of Ghana. Nevertheless, readers should note that this sample is not representative of the general female population in Ghana.

Third, the present study used a convenience sampling technique to recruit the participants. Convenience sampling is known to limit the generalizability of research results (i.e., external validity). Fourth, the present sample was recruited from the general female population in Ghana. Thus, the results may not apply to clinical female populations in Ghana (e.g., female survivors of breast cancer). Fifth, following the proposed modelling guidelines [56–59], this study relied heavily on ESEM with target rotation to validate the BCSBQ‐12 measure because the developers of the BCSBQ did not provide a theoretical basis for examining higher‐order (global) factors. However, future research may explore the possibility of a higher‐order factor model (i.e., bifactor CFA or bifactor ESEM) fitting the BCSBQ‐12 data. The interpretation of the present results should be guided by these limitations.

## 5. Conclusion

Despite its limitations, the present study provides strong evidence of construct validity and sound psychometric properties supporting the use of the BCSBQ‐12 for assessing breast cancer knowledge and screening beliefs among asymptomatic women in Ghana. That is, the key strengths of this study include the use of robust ESEM, the strong construct validity, and sound psychometric properties demonstrated by the Ghanaian data for the three‐factor BCSBQ‐12 model—an important contribution to the literature. In other words, the ESEM with target rotation supported the first‐order three‐factor oblique model proposed by the BCSBQ‐12, indicating the importance of considering breast cancer screening uptake from at least three key domains (i.e., *attitudes*, *knowledge,* and *barriers*). Further psychometric studies on the BCSBQ‐12 are needed to corroborate the present results.

**Nomenclature**
CFA: confirmatory factor analysis
EFA: exploratory factor analysis
ESEM: exploratory structural equation modelling
BCSBQ: Breast Cancer Screening Beliefs Questionnaire
WHO: World Health Organization
NHIS: National Health Insurance Scheme
ICCP: International Cancer Control Partnership
UGMC: University of Ghana Medical Centre Ltd
OSF: Open Science Framework

## Funding

This work was generously supported by the Andrew W. Mellon Foundation through the BECHS‐Africa Research Residency Programme.

## Disclosure

The author completed the residency at the American University in Cairo, Egypt, during which this paper was written. The views and conclusions expressed herein are solely those of the author and do not necessarily reflect those of The Andrew W. Mellon Foundation.

## Conflicts of Interest

The author declares no conflicts of interest.

## Supporting information


**Supporting Information** Additional supporting information can be found online in the Supporting Information section. Refer to the Supporting Information in the OSF repository for additional details related to this paper. Table S1: The 12‐item Breast Cancer Screening Beliefs Questionnaire. File S2: Raw dataset used in the present analysis (SPSS file). Table S3: Mplus syntax used for the model estimation.

## Data Availability

The data supporting the findings of this study are openly available in the OSF repository at https://doi.org/10.17605/OSF.IO/WMEAH. In this repository, I have provided the full dataset, M*plus* syntax used for model estimation, and the 12‐item BCSBQ.
